# Statistical Analysis of Community RNA Transcripts between Organic Carbon and Geogas-Fed Continental Deep Biosphere Groundwaters

**DOI:** 10.1128/mBio.01470-19

**Published:** 2019-08-13

**Authors:** Margarita Lopez-Fernandez, Elias Broman, Domenico Simone, Stefan Bertilsson, Mark Dopson

**Affiliations:** a Centre for Ecology and Evolution in Microbial Model Systems (EEMiS), Linnaeus University, Kalmar, Sweden; b SLU Bioinformatics Infrastructure, Swedish University of Agricultural Sciences, Uppsala, Sweden; c Department of Ecology and Genetics, Limnology and Science for Life Laboratory, Uppsala University, Uppsala, Sweden; d Department of Aquatic Sciences and Assessment, Swedish University of Agricultural Sciences, Uppsala, Sweden; CEH-Oxford

**Keywords:** deep biosphere, groundwaters, metatranscriptomes, protein-coding RNA, rRNA

## Abstract

Life in water-filled bedrock fissures in the continental deep biosphere is broadly constrained by energy and nutrient availability. Although these communities are alive, robust studies comparing active populations and metabolic processes across deep aquifers are lacking. This study analyzed three oligotrophic Fennoscandian Shield groundwaters, two “modern marine” waters that are replenished with organic carbon from the Baltic Sea and are likely less than 20 years old (171.3 and 415.4 m below sea level) and an extremely oligotrophic “thoroughly mixed” water (448.8 m below sea level) of unknown age that is composed of very old saline and marine waters. Cells were captured either using a sampling device that rapidly fixed RNA under *in situ* conditions or by filtering flowing groundwater over an extended period before fixation. Comparison of metatranscriptomes between the methods showed statistically similar transcript profiles for the respective water types, and they were analyzed as biological replicates. Study of the small subunit (SSU) rRNA confirmed active populations from all three domains of life, with many potentially novel unclassified populations present. Statistically supported differences between communities included heterotrophic sulfate-reducing bacteria in the modern marine water at 171.3 m below sea level that has a higher organic carbon content than do largely autotrophic populations in the H_2_- and CO_2_-fed thoroughly mixed water. While this modern marine water had signatures of methanogenesis, syntrophic populations were predominantly in the thoroughly mixed water. The study provides a first statistical evaluation of differences in the active microbial communities in groundwaters differentially fed by organic carbon or “geogases.”

## Abstract (precis)

Despite being separated from the photosynthesis-driven surface by both distance and time, the deep biosphere is an important driver for the earth’s carbon and energy cycles. However, due to the difficulties in gaining access and low cell numbers, robust statistical omics studies have not been carried out, and this limits the conclusions that can be drawn. This study benchmarks the use of two separate sampling systems and demonstrates that they provide statistically similar RNA transcript profiles, importantly validating several previously published studies. The generated data are analyzed to identify statistically valid differences in active microbial community members and metabolic processes. The results highlight contrasting taxa and growth strategies in the modern marine waters that are influenced by recent infiltration of Baltic Sea water versus the hydrogen- and carbon dioxide-fed, extremely oligotrophic, thoroughly mixed water.

## OBSERVATION

The deep biosphere is the largest biome on earth, where the continental subsurface alone hosts up to 6 × 10^29^ cells from all three domains (1). Deep life has been demonstrated as active by, e.g., “viable/dead” PCR amplification (2), “omics” (3–5), and video evidence (6). A previous study at the Swedish Nuclear Fuel and Waste Management Company (SKB)-operated Äspö Hard Rock Laboratory (Äspö HRL) used a specially designed sampling device to fix cells under *in situ* conditions to ensure that RNA transcripts were unaffected by sampling procedures (3). In contrast, other studies used cell capture from flowing groundwater on filters over several days prior to fixation (see, e.g., reference 4). However, it is unknown if extended capture times alter the RNA transcript profile.

The extreme oligotrophy in the continental deep biosphere can limit cell numbers to 10^1^ to 10^7^ cells/ml (1), while Äspö HRL groundwaters contain 10^5^ to 10^6^ cells/ml (7). Due to the difficulty of obtaining deep biosphere samples and the large water volume needed to extract sufficient RNA for sequencing, no omics studies have provided sufficient replicates for valid statistics.

In this study, we combined RNA transcript data from the sampling device (3) and from cells captured over several days on filter holders to evaluate if the two methods are comparable (see File S1 in the supplemental material). Additionally, we statistically analyzed gene transcript counts pertaining to active microbial taxa and their metabolic processes between groundwaters of various ages and origins.

10.1128/mBio.01470-19.1 FILE S1 Additional methods describing the sampled groundwaters, cell capture and fixation, negative controls and quality assurance, RNA sequencing, and bioinformatics plus statistical analyses. Download File S1, DOCX file, 0.1 MB. Copyright © 2019 Lopez-Fernandez et al. 2019 Lopez-Fernandez et al. This content is distributed under the terms of the Creative Commons Attribution 4.0 International license.

**FIG 1:**
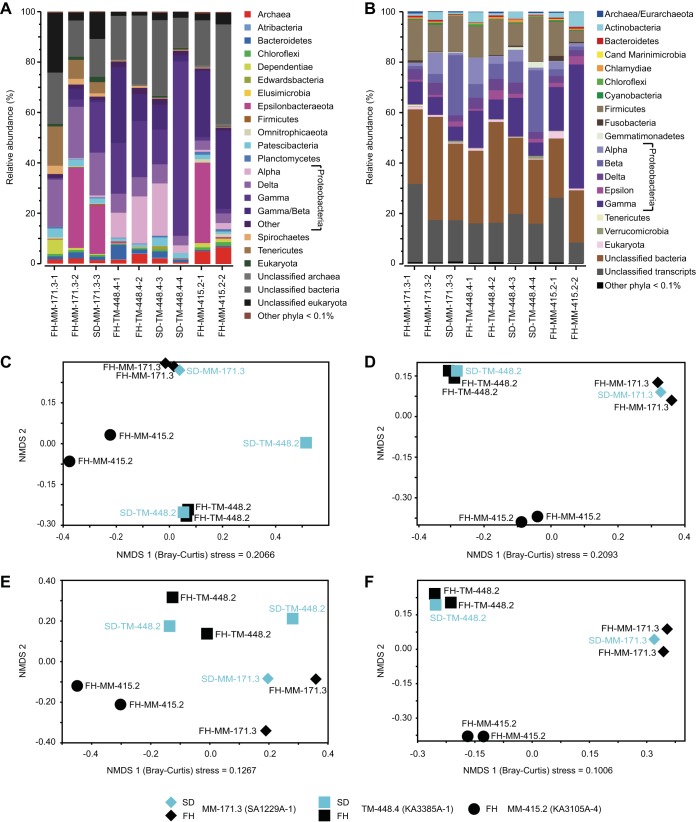
(A and B) Taxonomic annotation of the SSU rRNA (A) and protein-coding RNA (B) sequences showing stacked bars of the taxonomic phyla and *Proteobacteria* classes (*Betaproteobacteriales* shown separately) with a relative abundance of >0.1% for the modern marine (MM-171.3 and MM-415.2) and thoroughly mixed (TM-448.4) groundwaters. Rare taxa with a relative abundance of <0.1% are given as “other phyla.” (C and D) NMDS Bray-Curtis dissimilarity (beta diversity) plots based on the SSU rRNA at the lowest taxonomic level that could be assigned to the SILVA database using the Ribosomal Database Project classifier (C) and a second NMDS without the SD TM-448.3-4 (KA3385A-1) outlier (D). (E and F) NMDS Bray-Curtis plots based on the annotated transcripts (i.e., UniProtKB identifiers) with an E value of <10 and TPM of >100 from the full data set (E) and without the SD TM-448.4-4 outlier (F). The sampling methods are filter holders (FH) and sampling device (SD). Cand, *Candidatus*.

The studied groundwaters were two modern marine waters (MM-171.3 and MM-415.2) that are replenished from the Baltic Sea and have a residence time of <20 years and a “thoroughly mixed” water (TM-448.4) that is composed of different waters of multiple origins and unknown age (3, 7, 8). Cells were captured, and community RNA was extracted and sequenced according to File S1. The small subunit (SSU) rRNA sequences (File S2) were annotated against the SILVA database and normalized as relative abundances (File S3). The MM-415.2 filter holder metatranscriptomes only had two replicates and thus cannot be statistically compared to the others. However, this groundwater was clearly different in both its SSU and protein-coding RNA (pcRNA) transcripts (Fig. 1) and is discussed in File S4. Nonmetric multidimensional scaling (NMDS) of SSU rRNA transcript beta diversity suggested that the three water samples were statistically different in their microbial communities (permutational multivariate analysis of variance [PERMANOVA] 9,999 permutations, *P = *0.0011; Fig. 1). Previous analysis of the sampling device (SD) TM-448.4-4 sample showed it was different from the SD TM-448.4-3 sample, as it had likely been recently exposed to an electron donor (3). Repetition of the NMDS without this outlier altered the significance between the three groundwaters (*P = *0.004). Without TM-448.4-4, the grouping supports the notion that (i) the two methods give highly similar RNA transcript patterns and, therefore, sampling with filter holders over several days is valid, and (ii) in the absence of periodic availability of an electron donor (as for the SD TM-448.4-4 sample [3]), the deep biosphere communities were stable for a minimum of 2 years.

10.1128/mBio.01470-19.2 FILE S2 The upper table shows the amount of sequences received from the sequencing facility, after quality trimming, and the amount of small subunit reads extracted from the data set. The lower table shows the amount of transcripts (i.e., contigs) constructed during the *de novo* coassembly and the lengths of the contigs. Download File S2, XLSX file, 0.1 MB. Copyright © 2019 Lopez-Fernandez et al. 2019 Lopez-Fernandez et al. This content is distributed under the terms of the Creative Commons Attribution 4.0 International license.

10.1128/mBio.01470-19.3 FILE S3 Results from the annotation of SSU rRNA reads against the SILVA database. Values are shown as relative proportion [(*x*/sum) × 100]. Download File S3, XLSX file, 0.2 MB. Copyright © 2019 Lopez-Fernandez et al. 2019 Lopez-Fernandez et al. This content is distributed under the terms of the Creative Commons Attribution 4.0 International license.

10.1128/mBio.01470-19.4 FILE S4 Additional discussion of the MM-415.2 metatranscriptomes. Download File S4, DOCX file, 0.1 MB. Copyright © 2019 Lopez-Fernandez et al. 2019 Lopez-Fernandez et al. This content is distributed under the terms of the Creative Commons Attribution 4.0 International license.

SSU rRNA-based phylogeny from all analyzed metatranscriptomes showed that a broad range of phyla from all three domains of life were viable and had protein-synthesizing potential (3) (Fig. 1). It also reinforced that the deep biosphere contains a large relative proportion of active candidate phyla from all three domains (e.g., *Patescibacteria*) along with many unclassified sequences. Statistically valid differences between the MM-171.3 and TM-448.4 groundwaters included sulfate-reducing bacteria (SRB) with *Desulfobulbaceae* in the MM-171.3 groundwater compared to *Desulfobacteraceae* and Desulfurivibrio in the TM-448.4 groundwater (File S5). This confirms that sulfur compound reduction is prevalent (see, e.g., references 9 and 10) with the predominantly organoheterotrophic SRB *Desulfobulbaceae* (11) in the MM-171.3 groundwater compared to autotrophic *Desulfurivibrio* spp. (12) in the ultraoligotrophic TM-448.4 water. In addition, increased 16S rRNA gene transcripts in the TM-448.4 groundwater that aligned within the *Syntrophus* genus demonstrated that syntrophy is likely to be an important survival strategy in these oligotrophic groundwaters (13).

10.1128/mBio.01470-19.5 FILE S5 One-way analysis of variance (ANOVA) *post hoc* Tukey test of the difference between the 16S rRNA gene relative abundances (%) between the TM-448.4 and MM-171.3 (*n *=* *3 for each water type). Only taxa that are statistically different are shown. Download File S5, XLSX file, 0.1 MB. Copyright © 2019 Lopez-Fernandez et al. 2019 Lopez-Fernandez et al. This content is distributed under the terms of the Creative Commons Attribution 4.0 International license.

Analysis of pcRNA transcripts identified 973 unique prokaryote genes (File S6). The NMDS analysis also showed that the community-level transcription profiles were statistically different (*P = *0.002; Fig. 1), and further removal of the SD TM-448.4-4 outlier gave a *P *value of 0.004. Altogether, 410 prokaryotic genes had significant differential expression between the MM-171.3 and TM-448.4 groundwaters (false-discovery rate [FDR] < 0.05; E value < 0.001). Transcripts encoding tricarboxylic acid (TCA) cycle (*mdh, fumC*, and *sucC*) and ATP synthase (*atpAG*) proteins had higher transcript counts in the MM-171.3 groundwater, while increased TM-448.4 transcripts encoded, e.g., ribosomal (e.g., *rpmB*, *rpsBK*, and *rplC*) and stress/repair (e.g., *dfx*, *recGN*, *cspAB*, *clpPX*, *dnaK*, and *hspC4*) proteins. Additionally, a qualitative comparison of the SD TM-448.4-4 outlier (3) with the other three replicates suggested that this outlier had more transcripts involved with, e.g., replication and metabolic processes. Overall, most overexpressed transcripts were seen in the MM-171.3 groundwater, robustly demonstrating that this community was actively growing while the TM-448.4 populations were in “metabolic standby” (3).

10.1128/mBio.01470-19.6 FILE S6 Results from the annotation (*de novo* assembly) of the protein-coding RNA transcripts. Units are shown as transcripts per million sequences (TPM). Statistically significant protein-coding RNA transcripts for prokaryotes (FDR, <0.05; E value, <0.001 and then merged based on similar UniProtKB identifiers [IDs]) were tested between the MM-171.3 and TM-448.4 groundwaters. Download File S6, XLSX file, 0.4 MB. Copyright © 2019 Lopez-Fernandez et al. 2019 Lopez-Fernandez et al. This content is distributed under the terms of the Creative Commons Attribution 4.0 International license.

**FIG 2:**
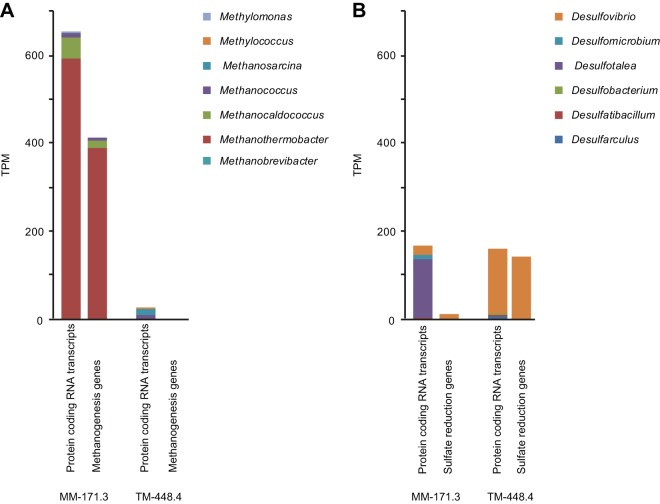
(A and B) Average of the significantly different (false-discovery rate [FDR] < 0.05; E value < 0.001) transcripts per million sequences (TPM) for the modern marine (MM-171.3) and thoroughly mixed (TM-448.4) groundwaters for protein-coding RNA transcripts assigned to methanogenic taxa (left) and genes attributed to methanogenesis from CO_2_ (*fwdC*, *mtrACDEH*, *mcrABCG*; right) (A), as well as protein-coding RNA transcripts assigned to sulfate-reducing bacteria taxa (left) and genes attributed to sulfate reduction (cytochrome *c*_3_, *rd2*, *aprA*, *dsrA*, and *dsvAB*; right) (B).

The metabolic process with the greatest number of statistically different MM-171.3 groundwater transcripts was methanogenesis from CO_2_ (*fwdC*, *mtrACDEH*, and *mcrABCG* genes) attributed to Methanothermobacter spp. (14) within the *Euryarchaeota* (Fig. 2 and File S7 and S8). Sulfur oxidation coupled to nitrate reduction was also important with increased pcRNA transcripts attributed to Sulfurimonas denitrificans (15) and Thiobacillus denitrificans (16) in the MM-171.3 and TM-448.4 groundwaters, respectively (File S9). This difference was potentially because S. denitrificans can use, e.g., formate, while *T. denitrificans* is an obligate sulfur compound-oxidizing chemolithoautotroph that had statistically increased *cbbLS* transcripts encoding CO_2_ fixation via the Calvin-Benson-Bassham cycle. Consistent with the SSU rRNA data, the pcRNA transcripts had significant differences in the SRB. These included transcripts from 37 genes attributed to the *Desulfobulbaceae* that were only present in the MM-171.3 water, while dissimilatory sulfate-reducing genes *aprA*, *dsrA*, and *dsvAB* attributed to *Desulfovibrio* spp. were increased in the TM-448.4 groundwater (Fig. 2 and File S8). The importance of syntrophy was also further demonstrated by pcRNA transcripts in both the MM-171.3 and TM-448.4 waters attributed to Syntrophus aciditrophicus that grows alongside H_2_ utilizers (17) predominantly present in the TM-448.4 groundwater (File S9). Finally, earlier observations of cyanobacteria in ancient deep terrestrial groundwaters (18, 19) were confirmed by increased Synechocystis pcRNA transcripts in the TM-448.4 water, also demonstrating their viability in these habitats.

10.1128/mBio.01470-19.7 FILE S7 Breakdown of the archaeal domain phyla from Fig. 1 showing relative proportions of the taxonomic annotation of the SSU rRNA. Download FILE S7, EPS file, 0.5 MB. Copyright © 2019 Lopez-Fernandez et al. 2019 Lopez-Fernandez et al. This content is distributed under the terms of the Creative Commons Attribution 4.0 International license.

10.1128/mBio.01470-19.8 FILE S8 Summary of the data shown in Fig. 2, including standard deviation. Breakdown of the average of the significantly different (false-discovery rate [FDR], <0.05; E value, <0.001) transcripts per million (TPM) sequences for the modern marine (MM-171.3) and thoroughly mixed (TM-448.4) groundwaters for protein-coding RNA transcripts assigned to methanogenic taxa and genes attributed to methanogenesis from CO_2_ (*fwdC*, *mtrACDEH*, *mcrABCG*), as well as protein-coding RNA transcripts assigned to sulfate-reducing bacteria taxa (left) and genes attributed to sulfate reduction (cytochrome *c*_3_, *rd2*, *aprA*, *dsrA*, and *dsvAB*) represented in stacked bars. Download FILE S8, XLSX file, 0.1 MB. Copyright © 2019 Lopez-Fernandez et al. 2019 Lopez-Fernandez et al. This content is distributed under the terms of the Creative Commons Attribution 4.0 International license.

10.1128/mBio.01470-19.9 FILE S9 Average TPM sequences in the three replicates of the modern marine (MM-171.3) and thoroughly mixed (TM-448.4) waters for all protein-coding genes assigned to the taxa *Sulfurimonas*, *Thiobacillus*, *Cyanobacteria*, and *Syntrophus*. Download FILE S9, EPS file, 1.9 MB. Copyright © 2019 Lopez-Fernandez et al. 2019 Lopez-Fernandez et al. This content is distributed under the terms of the Creative Commons Attribution 4.0 International license.

This work presents for the first time a statistically robust omics study of deep subsurface crystalline rock groundwaters with different depths and geochemical characteristics. We conclude that cell capture over several days does not alter RNA transcript profiles compared to rapid *in situ* fixation in this extremely oligotrophic environment. Importantly, this analysis of the two methods validates published studies that have used capture times prior to RNA fixation over the several days needed to obtain sufficient biomass for biomolecule extraction from low-cell-density deep groundwaters. The similarity of the data obtained by the two methods was likely due to the long-term and stable oligotrophic conditions in the respective groundwaters. These novel findings also provide evidence on how the differences in active communities and metabolic processes are influenced by organic carbon versus geogas-fed modern marine and thoroughly mixed groundwaters, respectively. This benchmarking of deep biosphere metatranscriptome analyses paves the way for future and still-needed exploration of the living deep biosphere in a statistically sound way.

### Data availability.

The raw sequence data are available in the NCBI Sequence Read Archive BioProject numbers PRJNA400688 and PRJNA541524 for the sampling device and filter holders, respectively.
