# Supplementary material for: Statistical Analysis of Community RNA Transcripts between Organic Carbon and Geogas-Fed Continental Deep Biosphere Groundwaters
Source: mBio. 2019 Aug 13;10(4):e01470-19. doi: 10.1128/mBio.01470-19 (PMC6692508; doi:10.1128/mBio.01470-19)
Supplement: FILE S1 [file mBio.01470-19-s0001.docx]

Statistical analysis of community RNA transcripts between organic carbon and ‘geogas’ fed continental deep biosphere groundwaters

Margarita Lopez-Fernandez^1^, Elias Broman^1^, Domenico Simone^1,2^, Stefan Bertilsson^3^, Mark Dopson^1^

^1^Centre for Ecology and Evolution in Microbial Model Systems (EEMiS), Linnaeus University, Stuvaregatan 4, 391 82 Kalmar, Sweden

^2^SLU Bioinformatics Infrastructure, Swedish University of Agricultural Sciences, Almas Allé 5, 750 07 Uppsala, Sweden

^3^Department of Ecology and Genetics, Limnology and Science for Life Laboratory, Uppsala University, Norbyvägen 18D, 752 36 Uppsala, Sweden

**Supplemental File 1.** Supplemental methods

**Description of the sampled groundwaters.** Three groundwaters containing ferrous iron, dissolved sulfide (HS^−^), temporally stable chemistry and δ^18^O, and neutral pH ([1](#_ENREF_1)) were sampled for this study: borehole SA1229A-1, KA3105A-4, and KA3385A-1. Borehole SA1229A-1 is located at 171.3 m below the sea level (mbsl) and this groundwater has tracers of marine waters ([2](#_ENREF_2)) and chemical values similar to that of modern Baltic Sea water ([3](#_ENREF_3)). Although the age of this groundwater is not known, it was estimated to be < 20 years ([4](#_ENREF_4)) and therefore it was termed ‘modern marine’ (‘MM-171.3’). The KA3105A-4 groundwater (located at 415.2 mbsl) is similar to MM-171.3 and accordingly, it was termed ‘MM-415.2’. Finally borehole KA3385A-1 (located at 448.4 mbsl) is a very old saline groundwater with a low chloride concentration as it had been diluted by waters with lower salinity a few thousands of years ago ([3](#_ENREF_3)). Therefore, it was termed ‘thoroughly mixed’ (‘TM-448.4’).

**Cell capture and fixation.** The three groundwaters were sampled under *in situ* conditions using two different sampling methods. Firstly, by connecting a sampling device with an in-built fixation system as described in Lopez-Fernandez et al. ([5](#_ENREF_5)) from June 2015 to March 2016, concretely on average a filter per day was collected for sample SD-MM-171.3-3 from 18/06/2015 to 07/08/2015, for sample SD-MM-171.3-4 from 07/03/2016 to 23/03/2016, for sample SD-TM-448.4-3 from 18/08/2015 to 17/09/2015, and for sample SD-TM-448.4-4 from 08/10/2015 to 04/02/2016. Secondly, by connecting a high-pressure stainless steel filter holder (Merck Millipore, USA) with a downstream needle valve and pressure gauge as described in Lopez-Fernandez et al. ([6](#_ENREF_6)) from September 2015 to December 2015. Specifically filters used for sample FH-MM-171.3-1 were collected on 07 and 14/09/2015, for sample FH-MM-171.3-2 on 09/11, 30/11 and 04/12/2015, for sample FH-MM-415.2-1 filters were collected on 25/06, 07/09 and 14/09/2015, for sample FH-MM-415.2-2 on 30/09, 02, 05 and 07/10/2015, for sample FH-TM-448.4-1 on 16/06/2015, and for sample FH-TM-448.4-2 on 04/12/2015. In both cases, borehole water was flushed for five section volumes to discard stagnant borehole water before collecting planktonic cells on sterile hydrophilic polyvinylidene fluoride (PVDF) membranes with 0.1 µm poresize (47 mm Durapore, Merck Millipore, USA) under *in situ* conditions. The cell collection using the sampling device was performed as previously described ([5](#_ENREF_5)). For the planktonic cell capture by using the filter holders an appropriate volume of groundwater was filtered before the filter was aseptically placed in a sterile Petri dish where cells were immediately fixed by adding 1 mL of a stop solution consisting of 5% (vol/vol) water-saturated phenol in absolute ethanol ([7](#_ENREF_7)). Afterwards, the filter was rolled and placed in a sterile cryogenic tube (Thermo Scientific, USA) and immediately frozen in liquid nitrogen and stored at – 80°C until further processing. The samples that were collected are described in the table below.

| Sample  ID | Borehole | Sampling  method | Water  type | Total water  volume (L) | RNA  (ng/µL) | cDNA  (ng/µL) |
| --- | --- | --- | --- | --- | --- | --- |
| SD-MM-171.3-3 | SA1229A-1 | Sampling device | Modern marine | 180 | 0.02 | 60 |
| SD-TM-448.4 -3 | KA3385A-1 | Sampling device | Thoroughly mixed | 140 | 0.02 | 39 |
| SD-TM-448.4-4 | KA3385A-1 | Sampling device | Thoroughly mixed | 150 | 0.02 | 33 |
|  |  |  |  |  |  |  |
| FH-MM-171.3-1 | SA1229A-1 | Filter holder | Modern marine | 177 | 17.3 | 42 |
| FH-MM-171.3-2 | SA1229A-1 | Filter holder | Modern marine | 295 | 6.5 | 57 |
| FH-MM-415.2-1 | KA3105A-4 | Filter holder | Modern marine | 337 | 11.9 | 60 |
| FH-MM-415.2-2 | KA3105A-4 | Filter holder | Modern marine | 295 | 12.6 | 60 |
| FH-TM-448.4-1 | KA3385A-1 | Filter holder | Thoroughly mixed | 635 | 4 | 60 |
| FH-TM-448.4-1 | KA3385A-1 | Filter holder | Thoroughly mixed | 625 | 6 | 59 |

**RNA extraction, cDNA generation, and negative controls.** RNA was extracted from all filters using the MO BIO PowerWater RNA isolation kit and used to generate cDNA using the Ovation^®^ RNA-Seq System V2 (NuGEN) as previously described ([5](#_ENREF_5)).

Negative and quality controls were performed including 1) RNA extraction from the MO BIO PowerWater RNA and Ovation^®^ RNA-Seq System V2 reagents. 2) RNA extraction and cDNA amplification from a sterile filter that was collected under the same conditions as for the fixed samples. 3) DNA contamination of the RNA extractions (after DNase treatment) was checked with 40 cycles of PCR amplification. All controls were negative as nucleic acid concentrations were below the Qubit 2.0 Fluorometer’s detection limit and no bands from cDNA amplification products were obtained after electrophoresis. All these controls support that the metatranscriptome sequencing was a true representation of the RNA transcripts within the community.

**Metatranscriptome library construction and sequencing.** cDNA library preparation and sequencing of all samples were performed at the Science for Life Laboratory, Sweden (www.scilifelab.se). Library preparation was carried out using the Illumina TruSeq Nano DNA Library Prep Kit for NeoPrep. Clustering was done by 'cBot' and samples were sequenced on HiSeq2500 (HiSeq Control Software 2.2.58/RTA 1.18.64) with a 2 × 126 bp setup using 'HiSeq SBS Kit v4' chemistry. The Bcl to FastQ conversion was performed using bcl2fastq-1.8.4 from the CASAVA software suite. The quality scale used was Sanger / phred33 / Illumina 1.8+.

**Bioinformatics and statistical analyses.** The samples retrieved from the sequence facility had on average 45.3 million read pairs. Pair end sequences were trimmed for Illumina adapters and low quality sequences using trimmomatic 0.32 ([8](#_ENREF_8)), and merged with FLASH v1.2.11 ([9](#_ENREF_9)) with the following settings: LEADING:20 TRAILING:20 SLIDINGWINDOW:4:25 MINLEN:100. The quality-filtered data yielded on average 34.7 million sequences (merged reads, and leftover non-combined pairs) with a 155 bp length per sample. SortMeRNA 2.1b in conjunction with the SSU SILVA 132 database was used with the merged pairs (as a single-end dataset) and leftover non-combined pairs (as paired-end dataset; R1 and R2) to extract archaeal, bacterial and eukaryotic small-subunit (SSU) rRNA sequences. This resulted in an average of 13.5 million 155 bp sequences per sample (on average ~40% SSU rRNA reads per sample; Supplemental File S2). The SSU rRNA sequences were then annotated against the SSU SILVA 132 Ref NR 99 database using RDP classifier 2.11 ([10](#_ENREF_10)) with default settings. Final counts were normalized among samples as relative proportions ((x/sum) × 100) and analyzed in Explicet 2.10.15 ([11](#_ENREF_11)). Non-metric multidimensional scaling (NMDS) plots were constructed in Past 3.17 and 3.19 ([12](#_ENREF_12)).

Merged pairs and non-combined pairs were used to construct a *de novo* co-assembly with the assembler Trinity 2.4.0 ([13](#_ENREF_13)) using the --single and --run_as_paired commands. This yielded 420 966 protein coding RNA transcripts with an average length of 317 bp. The PhiX sequence control genome was detected in the assembly, checked against the NCBI GenBank database, and removed from the assembly. Annotation and transcript counts were conducted according to the Trinotate pipeline with default settings (<https://trinotate.github.io/>). In more detail, the co-assembly was annotated with BLASTX 2.6.0+ ([14](#_ENREF_14)) against the UniProtKB/Swiss-Prot database. Estimation of counts per transcript was conducted with Bowtie2 2.3.2 ([15](#_ENREF_15)), SAMtools 1.5 ([16](#_ENREF_16)), and RSEM 1.2.29 ([17](#_ENREF_17)) using a single end setup with the merged reads and a paired-end setup with the uncombined pairs. Counts from the two datasets (merged and uncombined pairs) were summed together and transcript counts normalized within each sample as Transcripts per Kilobase Million counts (TPM) and then cross normalized between samples using the Trimmed Mean of M values method (TMM). Final counts were expressed as TMM TPM values (as default in the Trinotate pipeline). UniProtKB identifiers obtained from the annotation were delimited by e-values ≤ 0.001 and afterwards duplicate identifiers were merged and associated TPM values summed. The data was then analyzed further with the “Retrieve/ID mapping” tool on the official UniProtKB website to link gene symbols, protein names, GO categories, Kegg KO identifiers, and taxonomic affiliation (<http://www.uniprot.org/>). The taxonomy of the contigs reconstructed with the *de novo* co-assembly was determined by using Kaiju 1.5.0 ([18](#_ENREF_18)) against the Kaiju webserver NCBI BLAST nr +euk 2017-05-16 database. Differently abundant RNA transcripts between sites and sampling method (i.e. Filter Holder and Sampling Device) were tested with edgeR analysis ([19](#_ENREF_19)) by using the perl script “run_DE_analysis.pl” supplied with Trinity 2.8.2 ([20](#_ENREF_20)). The script inputs raw read data, and uses edgeR 3.24.3 R package to normalize counts, and perform differential gene expression analysis. False discovery rate (FDR) values < 0.05 was used as an indication of statistically significant differences.

**References**

1. Wu X, Holmfeldt K, Hubalek V, Lundin D, Åström M, Bertilsson S, et al. Microbial metagenomes from three aquifers in the Fennoscandian shield terrestrial deep biosphere reveal metabolic partitioning among populations. The ISME journal. 2015;10:1192-203.

2. Gimeno MJ, Auqué LF, Acero P, Gómez JB. Hydrogeochemical characterisation and modelling of groundwaters in a potential geological repository for spent nuclear fuel in crystalline rocks (Laxemar, Sweden). Appl Geochem. 2014;45:50-71.

3. Mathurin FA, Astrom ME, Laaksoharju M, Kalinowski BE, Tullborg EL. Effect of tunnel excavation on source and mixing of groundwater in a coastal granitoidic fracture network. Environ Sci Technol. 2012;46:12779-86.

4. Mathurin FA, Drake H, Tullborg E-L, Berger T, Peltola P, Kalinowski BE, et al. High cesium concentrations in groundwater in the upper 1.2 km of fractured crystalline rock – Influence of groundwater origin and secondary minerals. Geochim Cosmochim Acta. 2014;132:187-213.

5. Lopez-Fernandez M, Simone D, Wu X, Soler L, Nilsson E, Holmfeldt K, et al. Metatranscriptomes Reveal That All Three Domains of Life Are Active but Are Dominated by Bacteria in the Fennoscandian Crystalline Granitic Continental Deep Biosphere. mBio. 2018;9(6):e01792-18.

6. Lopez-Fernandez M, Åström M, Bertilsson S, Dopson M. Depth and dissolved organic carbon shape microbial communities in surface influenced but not ancient saline terrestrial aquifers. Frontiers in Microbiology. 2018;9:2880.

7. Feike J, Jürgens K, Hollibaugh JT, Krüger S, Jost G, Labrenz M. Measuring unbiased metatranscriptomics in suboxic waters of the central Baltic Sea using a new in situ fixation system. The ISME journal. 2012;6(2):461-70.

8. Bolger AM, Lohse M, Usadel B. Trimmomatic: A flexible trimmer for Illumina sequence data. Bioinformatics. 2014;30(15):2114–20.

9. Magoč T, Salzberg SL. FLASH: fast length adjustment of short reads to improve genome assemblies. Bioinformatics. 2011;27(21):2957-63.

10. Wang Q, Garrity GM, Tiedje JM, Cole JR. Naïve bayesian classifier for rapid assignment of rRNA sequences into the new vacterial taxonbmy. Appl Environ Microbiol. 2007;73(16):5261-7.

11. Robertson CE, Harris JK, Wagner BD, Granger D, Browne K, Tatem B, et al. Explicet: graphical user interface software for metadata-driven management, analysis and visualization of microbiome data. Bioinformatics. 2013;29(23):3100-1.

12. Hammer Ø, Harper DAT, Ryan PD. PAST: Paleontological statistics software package for education and data analysis. Palaeontologia Electronica. 2001;4(1):9.

13. Haas BJ, Papanicolaou A, Yassour M, Grabherr M, Blood PD, Bowden J, et al. De novo transcript sequence reconstruction from RNA-seq using the Trinity platform for reference generation and analysis. Nat Protoc. 2013;8(8):1494-512.

14. Altschul SF, Gish W, Miller W, Myers EW, Lipman DJ. Basic local alignment search tool. Journal of Molecular Biology. 1990;215(3):403-10.

15. Langmead B, Trapnell C, Pop M, Salzberg SL. Ultrafast and memory-efficient alignment of short DNA sequences to the human genome. Genome Biol. 2009;10(3):R25.

16. Li H, Handsaker B, Wysoker A, Fennell T, Ruan J, Homer N, et al. The sequence alignment/map format and SAMtools. Bioinformatics. 2009;25(16):2078-9.

17. Li B, Dewey C. RSEM: accurate transcript quantification from RNA-Seq data with or without a reference genome. BMC Bioinformatics. 2011;12(1):323.

18. Menzel P, Ng KL, Krogh A. Fast and sensitive taxonomic classification for metagenomics with Kaiju. Nature communications. 2016;7.

19. Haas BJ, Papanicolaou A, Yassour M, Grabherr M, Blood PD, Bowden J, et al. De novo transcript sequence reconstruction from RNA-seq using the Trinity platform for reference generation and analysis. Nature Protocols. 2013;8:1494.

20. Robinson MD, McCarthy DJ, Smyth GK. edgeR: a Bioconductor package for differential expression analysis of digital gene expression data. Bioinformatics (Oxford, England). 2010;26:139-40.
