# Supplementary material for: Statistical Analysis of Community RNA Transcripts between Organic Carbon and Geogas-Fed Continental Deep Biosphere Groundwaters
Source: mBio. 2019 Aug 13;10(4):e01470-19. doi: 10.1128/mBio.01470-19 (PMC6692508; doi:10.1128/mBio.01470-19)
Supplement: FILE S4 [file mBio.01470-19-s0004.docx]

Statistical analysis of community RNA transcripts between organic carbon and ‘geogas’ fed continental deep biosphere groundwaters

Margarita Lopez-Fernandez^1^, Elias Broman^1^, Domenico Simone^1,2^, Stefan Bertilsson^3^, Mark Dopson^1^

^1^Centre for Ecology and Evolution in Microbial Model Systems (EEMiS), Linnaeus University, Stuvaregatan 4, 391 82 Kalmar, Sweden

^2^SLU Bioinformatics Infrastructure, Swedish University of Agricultural Sciences, Almas Allé 5, 750 07 Uppsala, Sweden

^3^Department of Ecology and Genetics, Limnology and Science for Life Laboratory, Uppsala University, Norbyvägen 18D, 752 36 Uppsala, Sweden

**Supplemental File S4** Addition discussion of the SD-MM-415.2 metatranscriptomes

As for the other two groundwaters, the MM-415.2 SSU rRNA gene profiles contained many candidate and unclassified transcripts that demonstrate that there is a large diversity in the deep biosphere waiting to be characterized. The MM-415.2 filter holder metatranscriptomes had a large relative proportion of Betaproteobacteriales that are present in oligotrophic groundwaters and are viable at the Äspö HRL [1] as well as Epsilonbacteraeota that are typically involved in sulfur cycling [2]. In addition, the MM-415.2 active community had the highest proportion of Archaeal SSU rRNA sequences (Fig. 1) that were predominantly from Nanoarchaeota with smaller proportions of Diapherotrites (Supplemental File S5). These phyla are from the DPANN radiation that often have minimal genome sizes and are potential symbionts [3]. Many deep biosphere populations have smaller genomes than their closest surface relative does and this is suggested to be an adaptation to the ultra-oligotrophic conditions [4] and this trait is prevalent in the terrestrial subsurface [5].

The highest average number of protein coding RNA (pcRNA) transcripts in the two MM-415.2 metatranscriptomes encoded the antibiotic resistance oleandomycin glycosyltransferase (*oleD*) with 116 323 and 140 411 TPM in SD-MM-415.2-1 and SD-MM-415.2-2, respectively (Supplemental File S6). A second gene with high numbers of pcRNA transcripts is methylenetetrahydrofolate dehydrogenase (*lptD*) that aids in solvent resistance. Finally, in contrast to the suggestion that motility is lost as a survival strategy in extremely oligotrophic environments, the flagellar P-ring protein 1 gene (*flgI1*) had 884 and 591 TPM in MM-415.2-1 and MM-415.2-2, respectively.

**References**

1. Lopez-Fernandez M, Broman E, Wu X, Bertilsson S, Dopson M. Investigation of viable taxa in the deep terrestrial biosphere suggests high rates of nutrient recycling. FEMS Microbiol Ecol. 2018;94:doi: 10.1093/femsec/fiy121.

2. Waite DW, Vanwonterghem I, Rinke C, Parks DH, Zhang Y, Takai K, et al. Comparative genomic analysis of the class Epsilonproteobacteria and proposed reclassification to Epsilonbacteraeota (phyl. nov.). Front Microbiol. 2017;8:682.

3. Castelle CJ, Brown CT, Anantharaman K, Probst AJ, Huang RH, Banfield JF. Biosynthetic capacity, metabolic variety and unusual biology in the CPR and DPANN radiations. Nature Revi Microbiol. 2018;16:629-45.

4. Wu X, Holmfeldt K, Hubalek V, Lundin D, Åström M, Bertilsson S, et al. Microbial metagenomes from three aquifers in the Fennoscandian shield terrestrial deep biosphere reveal metabolic partitioning among populations. The ISME journal. 2015;10:1192-203.

5. Probst AJ, Ladd B, Jarett JK, Geller-McGrath DE, Sieber CMK, Emerson JB, et al. Differential depth distribution of microbial function and putative symbionts through sediment-hosted aquifers in the deep terrestrial subsurface. Nature Microbiol. 2018;3:328-36.
